# Understanding the Spatial Clustering of Severe Acute Respiratory Syndrome (SARS) in Hong Kong

**DOI:** 10.1289/ehp.7117

**Published:** 2004-07-27

**Authors:** P.C. Lai, C.M. Wong, A.J. Hedley, S.V. Lo, P.Y. Leung, J. Kong, G.M. Leung

**Affiliations:** ^1^Department of Geography, and; ^2^Department of Community Medicine, University of Hong Kong, Hong Kong Special Administrative Region, People’s Republic of China; ^3^Health Welfare and Food Bureau and; ^4^Department of Health, Hong Kong Special Administrative Region, People’s Republic of China; ^5^Division of Health Informatics, Hong Kong Hospital Authority, Hong Kong Special Administrative Region, People’s Republic of China

**Keywords:** geographic information systems, GIS, SARS, severe acute respiratory syndrome, spatial distribution

## Abstract

We applied cartographic and geostatistical methods in analyzing the patterns of disease spread during the 2003 severe acute respiratory syndrome (SARS) outbreak in Hong Kong using geographic information system (GIS) technology. We analyzed an integrated database that contained clinical and personal details on all 1,755 patients confirmed to have SARS from 15 February to 22 June 2003. Elementary mapping of disease occurrences in space and time simultaneously revealed the geographic extent of spread throughout the territory. Statistical surfaces created by the kernel method confirmed that SARS cases were highly clustered and identified distinct disease “hot spots.” Contextual analysis of mean and standard deviation of different density classes indicated that the period from day 1 (18 February) through day 16 (6 March) was the prodrome of the epidemic, whereas days 86 (15 May) to 106 (4 June) marked the declining phase of the outbreak. Origin-and-destination plots showed the directional bias and radius of spread of superspreading events. Integration of GIS technology into routine field epidemiologic surveillance can offer a real-time quantitative method for identifying and tracking the geospatial spread of infectious diseases, as our experience with SARS has demonstrated.

Since the emergence and rapid spread of the etiologic agent of severe acute respiratory syndrome (SARS)—SARS coronavirus (SARS-CoV)—in late 2002 and during the first 6 months of 2003, great progress has been made in understanding the biology, pathogenesis, and epidemiology of both the disease and the virus (SARS-CoV). Much remains to be done, however, including the development of effective therapeutic interventions and diagnostic tools with high sensitivity and specificity soon after the onset of clinical symptoms. The evaluation of key epidemiologic parameters and the impact of different public health interventions in the various settings that experienced minor or major epidemics is also needed (Affonso et al. 2004; Cui et al. 2003; Lau et al. 2004; Leung et al., in press). In terms of outbreak control on the population level, many questions about “superspreading events” (SSEs) remain to be investigated. Such an SSE was responsible for > 300 cases (out of a total of 1,755) in the Amoy Garden Housing Estate (AMOY) in the Hong Kong epidemic. Moreover, Donnelly et al. (2003) have demonstrated that there were clear geographic concentrations of microclusters of SARS cases where the density of infection varied widely between different districts.

The application of geographic information system (GIS) methods in health and health care is a relatively new approach that started to gain acceptance a decade ago (Higgs and Gould 2001; Meade and Earickson 2000). In particular, a wide variety of cartographic methods have become available for the mapping and analysis of communicable disease data since the defining work of Cliff and Haggett (1988) and Haggett (1994). Advances in new technologies enable the application of GIS to examine spatially related problems from different perspectives. In addition to the descriptive mapping function, GIS possesses capabilities of data manipulation and geostatistical analysis.

In the present study, we applied GIS technology in mapping and visualizing the SARS outbreak in Hong Kong. In this article we focus on cartographic and geostatistical methods in representing and analyzing the patterns of disease spread during the 2003 outbreak. We also address the utility and limitations of GIS as a real-time disease surveillance tool.

## Materials and Methods

### Data sources.

We used spatial and nonspatial data in this study. Spatial data are geographic in nature and have a physical dimension or location in the real world. These are represented as points, lines, or area symbols, and they form the map base upon which SARS occurrences are depicted. Data on SARS incidence were derived from case-contact interviews that are text based; associated residential address data were first cleaned, checked for completeness and accuracy (e.g., Chinese-English transliteration of building and street names), and then geo-referenced to enable mapping.

We analyzed the SARSID integrated database (coordinated by the Department of Community Medicine, University of Hong Kong, on behalf of the Health, Welfare and Food Bureau—derived from the Hong Kong Hospital Authority eSARS system and the Department of Health’s Master List), which contained details on all patients confirmed to have SARS and admitted to hospitals in Hong Kong throughout the entire epidemic, that is, from 15 February to 22 June 2003. The criteria for inclusion in the SARSID were radiographic evidence of infiltrates consistent with pneumonia, fever ≥38°C or history of such at any time in the past 2 days, and at least two of the following: *a*) history of chills in the past 2 days; *b*) cough (new or increased cough) or breathing difficulty; *c*) general malaise or myalgia; and *d*) known history of exposure. However, patients were excluded if an alternative diagnosis could fully explain their illness. Moreover, each case classified as confirmed SARS was verified by the Hong Kong Department of Health according to World Health Organization (WHO) guidelines on case definitions (WHO 2003). Eighty-two percent of the 1,755 cases listed as confirmed SARS had either reverse transcription–polymerase chain reaction results positive for SARS-CoV or a 4-fold increase in IgG antibodies in paired sera (at admission and 21 or 28 days after symptom onset). Two questionnaires (case questionnaire and case-contact survey) were administered, mostly through telephone interviews, to all SARS cases confirmed by the Department of Health, initially by four regional field offices and later by a central interviewing team of nurses, to record symptoms at presentation to the hospital and to identify contacts and events of probable significance to transmission.

A total of 1,709 confirmed cases (out of 1,755 total cases) were extracted for the analysis. Forty-six cases (i.e., 2.6% of the total) could not be pinpointed at an exact location because of inconsistencies in the address entries (So 2002).

### Geostatistical analyses.

We carried out three levels of analysis: *a*) an elementary analysis involving simple visual inspection of a geographic phenomenon; *b*) a cluster analysis attempting the identification of possible “hot spots,” and *c*) a contextual analysis aiming to explain relationships among geographic phenomena (Bailey and Gatrell 1995; Olson 1976).

At the elementary level, the spread of a disease in a community is revealed through the plotting of disease occurrences at residential addresses of the patients enabled with the address matching function in a GIS. Point by point is the simplest form of mapping disease occurrences without accounting for the magnitude at each location, but the sheer number and spread of points could have impeded effective reading of the event. A map of cumulative counts collapses the numerous observations into circles of varying sizes to signify differences in the magnitude of disease occurrences in the community. The circles are proportionally sized to reflect the number of occurrences at the sites, and geographic clustering of disease infection can then be clearly identified.

We also examined the spread of SARS over time on the basis of point patterns. Each disease occurrence was plotted spatially and the spread or dispersion of disease incidence was examined using nearest neighbor analysis based on the *R* scale. The nearest neighbor analysis is an accepted spatial statistical analysis used by environmental scientists to study species distribution (Krebs 1989) and by crime analysts to explain the levels of dispersion in crime and disorder data (Eck and Weisburd 1995). The *R* scale assumes that events will be randomly spaced unless something influences the distribution. Three different patterns are possible: clustered (0 ≤*R* < 0.8), distributed randomly (0.8 ≤*R* < 1.8), or with uniform spacing (1.8 ≤*R* ≤2.149). A contagious process will give rise to a clustered pattern with near-zero *R* values.

Cluster analysis involves statistical mapping that generalizes the numerous observations into a statistical surface to highlight spatial variation. A 5-day incubation period, consistent with a previous gamma distribution parameter estimation exercise (Leung et al., in press), was used to restructure the data for a time-series study. A statistical surface was created by the kernel method (Bailey and Gatrell 1995) for each day to reveal daily changes of disease hot spots. A kernel size of 300 × 300 m^2^ was used to reconstruct the territory of Hong Kong into a gridded surface of 208 columns and 151 rows. The kernel size was 300 × 300 m^2^, and disease occurrences within a bandwidth of 600 m from the kernel were summarized to yield density measures in terms of number of SARS cases per square meter. Each grid was then designated either as urban or suburban based upon land use classification, and its associated density measure was adjusted for the underlying variation in population density (i.e., kernel density × population density × grid cell size/1,000) to yield infection rates per 1,000 population. We adopted the approach by Kafadar (1996) but modified it to account for variation between urban or suburban population densities within a given district in Hong Kong (Table 1). Each urban or suburban grid was considered a homogeneous unit wherein its population density was apportioned according to the proportion of residents in the employed labor force.

We created 12 kernel maps adjusted for population at risk to characterize changes in disease hot spots on 12 prototypical days over 16 weeks in a chronologic sequence. The infection rates, which span across a wide range, were collapsed into 15 classes to reduce the complexity of map representation. Each of the 15 classes was assigned a shade in proportion to the magnitudes, with darker shades representing higher densities of infection. Two kinds of indexes were employed to assess the extent of disease clustering: *R* scale and Moran’s *I* coefficient for more highly connected grids of the queen’s case that considers a neighborhood of eight cells in a 3 × 3 matrix. Moran’s *I* coefficient ranges between −1 and 1 and is interpreted as regionalized or juxtaposition of similar values (0.6 ≤*I* ≤1 indicating positive spatial autocorrelation), lack of autocorrelation, or the actual arrangement of values as one that we would expect from a random distribution (−0.6 < *I* < 0.6 indicating no spatial correlation), and either contrasting or tendency for dissimilar values to cluster (−1 ≤*I* ≤−0.6 indicating negative spatial correlation). Although *R* scale is a global measure for the spread or dispersion of disease incidence for point data based on nearest neighbor distance (Eck and Weisburd 1995; Krebs 1989; Taylor 1977), Moran’s coefficient measures local spatial autocorrelation for area data (Getis and Ord 1992; Sawada 2001). A comparison of the power evaluation of disease clustering tests has been described by Song and Kulldorff (2003).

For contextual analysis, histograms of the kernel data for 12 prototypical days were drawn to highlight variation in infection rates. Also, we replaced mean and SDs of the classed density data with their natural logarithm functions to accentuate the effect of change between near-zero values; we then graphed the values.

We also established a breakdown of disease occurrences by recognized clusters (e.g., SSEs) for contextual analysis. Three disease clusters each with > 30 observations were extracted: AMOY, Prince of Wales Hospital (PWH), and Lower Ngau Tau Kok Housing Estate (NTKLOW). These data were used to derive origin-and-destination (OD) plots or flow diagrams. Lines were drawn to connect an origin location where the flow started (e.g., index source of infection) with related destinations where the flow ended (e.g., residences of secondary contacts). The OD plots are an established methodology employed by transport professionals and human geographers to examine the extent of spatial interaction and human settlement, as well as the modeling of commodity flows (Batten and Boyce 1986). The flow data themselves can be people, goods, telecommunications, and so on. The lines help to delimit the spatial coverage revealing the extent or degree of spread. SD ellipses centered on the geometric mean of all locations were drawn to provide a summary trend of the dispersion and to examine whether a distribution has a directional bias. The major axis is the direction of maximum spread of the point events, and the minor axis is the direction of minimum spread.

All analyses were carried out using ArcGIS software and its extension modules (Environmental Systems Research Institute, Redlands, CA, USA).

## Results

### Elementary analysis.

**Figure 1:**
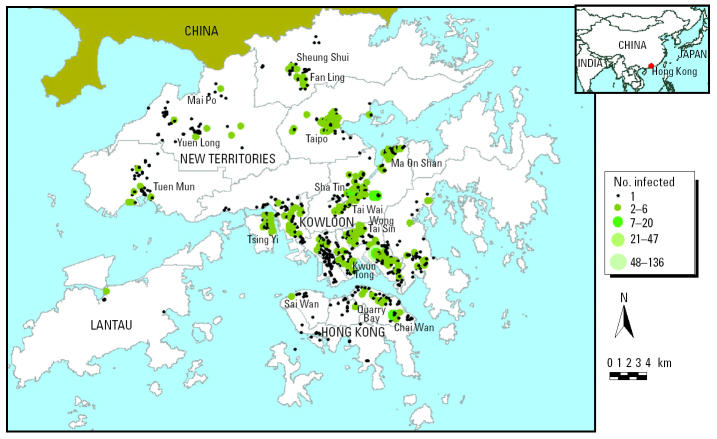
A summary map of SARS-infected cases in Hong Kong (February–June 2003). Data from the SARSID integrated database.

Figure 1 illustrates geographic locations of SARS infection by residential address in Hong Kong. The size of the circle corresponds to the density of cases in a particular location. There was clear clustering of cases in certain districts of the Kowloon peninsula (Kwun Tong, in which AMOY is located) and the New Territories (including Shatin, Tai Po), but Hong Kong Island was relatively spared. Table 2 supports this observation: most affected buildings or apartment blocks had very few cases, whereas seven buildings had > 10 SARS-affected patients.

### Cluster analysis.

**Figure 2:**
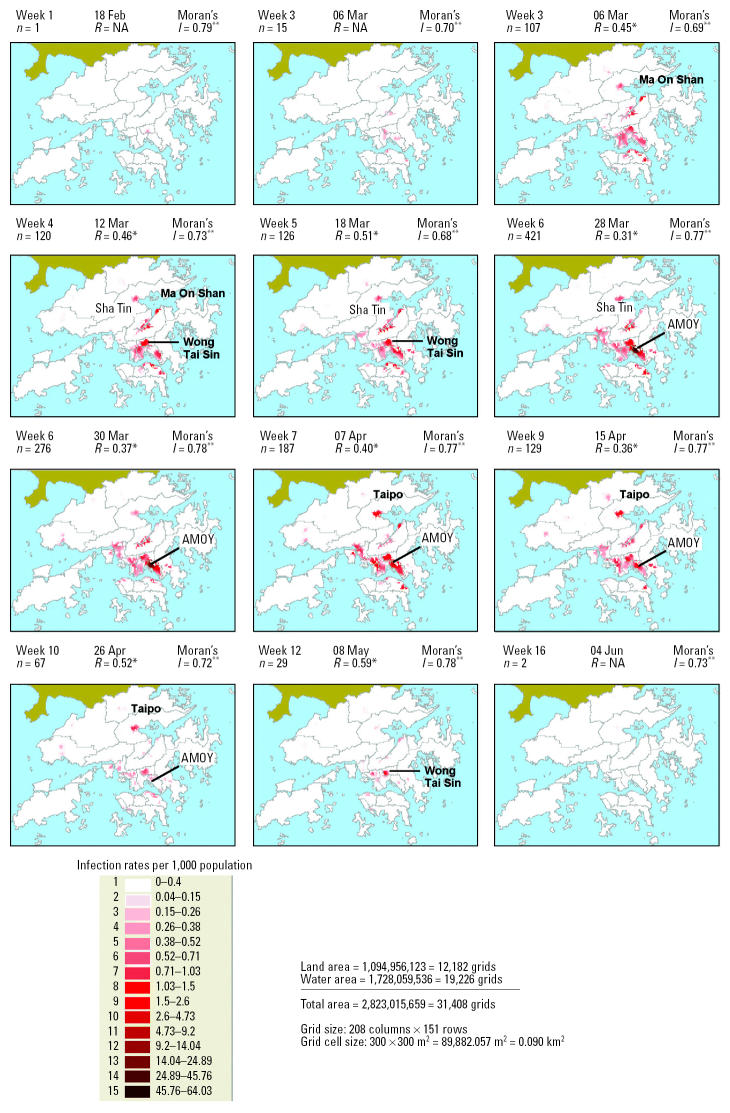
Time sequence of the spatial spread of SARS in Hong Kong (by date of onset with 5-day incubation period and weighted by population density), February–June 2003. Abbreviations: *n*, number of SARS patients; NA, not computed because of insufficient sample size (*n* < 25). An animated series is available online (Lai and Chan 2004).
*p < 0.01, which indicates a tendency toward clumping of disease incidence. **p < 0.001, which implies that spatial autocorrelation exists and that similar values on the map tend to cluster together.

**Figure 3:**
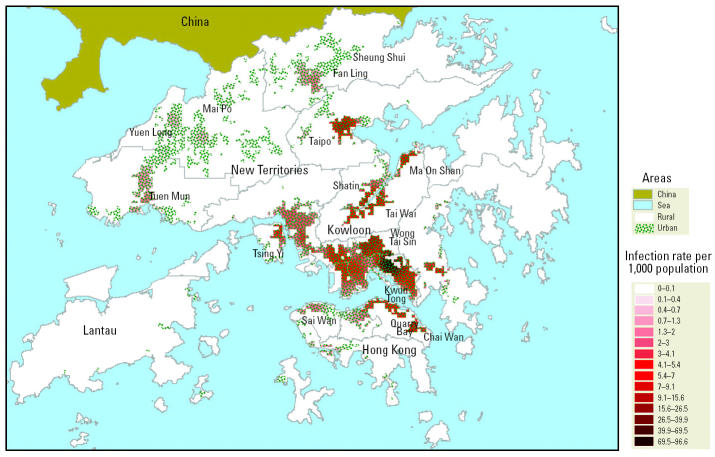
SARS hot spots based on cumulative disease occurrences from February through June 2003. Moran’s *I* = 0.78 (*p* < 0.001).

A series of 12 kernel maps based on date of symptom onset and accounting for a 5-day incubation period of SARS is presented in Figure 2. Each kernel map shows the density of SARS patients adjusted for underlying population density (i.e., SARS infection rate per 1,000 population) on a prototypical day over 16 weeks, with darker zones emphasizing disease hot spots [see also daily animated series by Lai and Chan (2004)]. A few disease hot spots were shown to be developing in the Kowloon peninsula and southeast New Territories (i.e., Ma On Shan and Shatin) by 10 March, which was followed later by a heavy concentration at the AMOY by 28 March. By early April, the AMOY case load began to dissipate and a new hot spot emerged in Tai Po (northeast New Territories). There is clear evidence of varying degrees of clustering as the epidemic progressed over time based on the low *R* values. The low *R* values signify substantial degrees of clustering (significant at 99% confidence level), with higher degrees of clustering occurring around the peak of the infection and relatively small divergences from random distribution at the beginning of the outbreak. High Moran’s *I* coefficients of ≥0.6 indicate that similar values tend to cluster together, which confirms the geospatial clustering and thus infectious nature of the disease, based on rates that were adjusted for the underlying population density. Figure 3 summarizes SARS hot spots in Hong Kong considering cumulative disease occurrences from February through June 2003. The map shows that the urban population was at higher risk of contracting SARS (Moran’s *I* = 0.78, *p* < 0.001), having already accounted for variation in population density.

### Contextual analysis.

**Figure 4:**
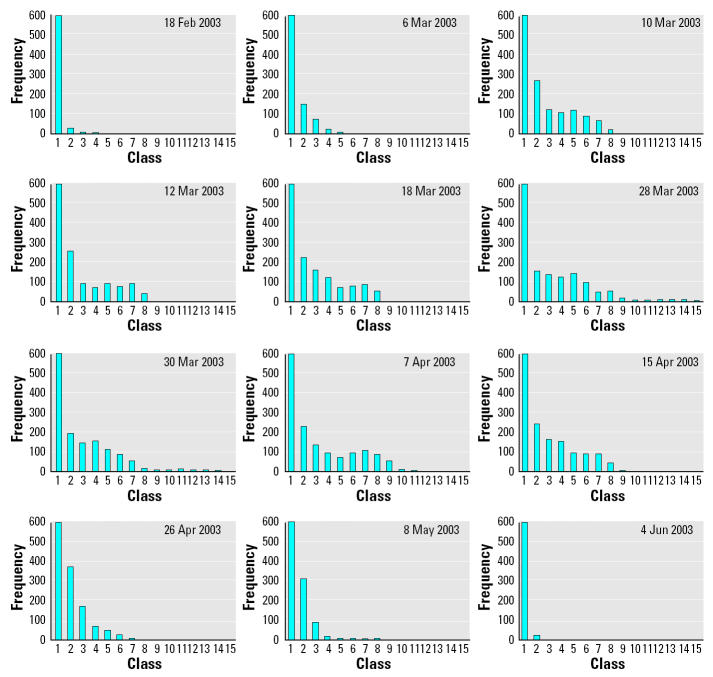
Daily histograms of SARS by classes on infection rates. Frequency counts are truncated at 600. Fifteen classes represent different ranges of infection rate per 1,000 population.

**Figure 5:**
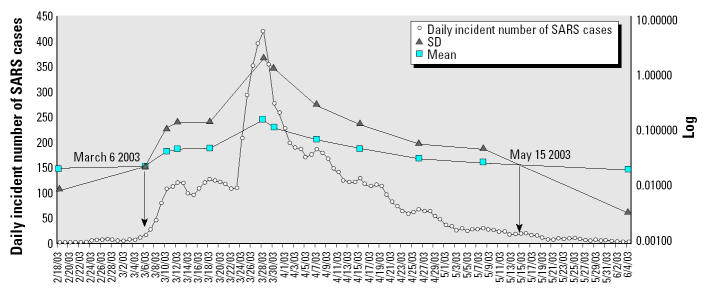
A logarithmic plot of mean and SD of infection rates of 12 prototypical days throughout the epidemic.

Daily histograms of the number of observations by 15 classes of infection rates, primarily composed of inverse J-shaped curves, show an increased concentration of SARS occurrences toward the end of March (Figure 4). Figure 5 is a logarithmic plot of the mean and SD of the infection rates of the 12 prototypical days representing different stages of the epidemic; values for individual days are presented in Table 3. Pairwise comparisons between each of the prototypical days and day 1 (or the day of indifference) of the epidemic demonstrated no detectable difference between the mean infection rates throughout the epidemic. However, there were statistically significant differences, by the *F*-test at a 0.01 significance level, in the SDs of the middle 10 prototypical days compared to day 1, suggesting unequal population variances during much of the outbreak. Higher *F*-values indicate more unequal variance. Given that the SD is a measure of geographic dispersion, we can infer that a larger SD signifies a wider spread of the disease over the territory. The crossover points of the mean and SD curves in Figure 5 indicate, on the one end, the beginning of substantial disease spread across the territory, and on the other end, the subsidence of the epidemic. Therefore, the time from day 1 (18 February) through day 16 (6 March) was the prodrome of the epidemic, whereas days 86 (15 May) through 106 (4 June) marked the declining phase of the outbreak.

**Figure 6:**
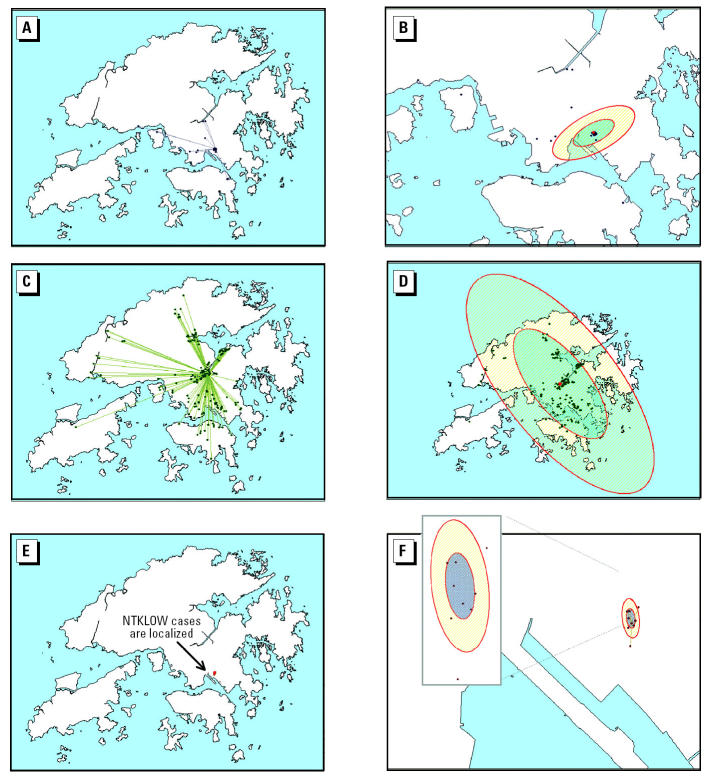
Extent and trend of spatial spread of known disease clusters. (*A*) AMOY cluster (*n* = 335; *R* = 0.15; *p* < 0.001); the null hypothesis of a random pattern is rejected and the point patterns exhibit a high tendency toward clustering. (*B*) SD ellipses for AMOY cluster (ellipse 1: *x*-length = 869.87, *y*-length = 2044.20; ellipse 2: *x*-length = 1739.74, *y*-length = 4088.40). (*C*) PWH cluster (*n* = 212; *R* = 0.45; *p* < 0.001); the null hypothesis of a random pattern is rejected, and the point patterns exhibit a tendency toward clustering but a more widespread distribution compared with the others. (*D*) SD ellipses for PWH cluster (ellipse 1: *x*-length = 7889.94, *y*-length = 18541.37; ellipse 2: *x*-length = 15779.88, *y*-length = 37082.74). (*E*) NTKLOW cluster (*n* = 38; *R* = 0.22; *p* < 0.001); the null hypothesis of a random pattern is rejected and the point patterns exhibit a high degree of clustering. (*F*) SD ellipses for NTKLOW cluster (ellipse 1: *x*-length = 59.34, *y*-length = 139.44; ellipse 2: *x*-length = 118.67, *y*-length = 278.88).

OD plots of disease clusters were obtained by linking patients’ places of residence with the likely or probable locations of index cases or environmental sources of infection as defined through contact tracing by public health authorities (Figure 6). PWH is a tertiary teaching hospital and the site of the first SSE and nosocomial cluster in the Hong Kong epidemic, whereas AMOY and NTKLOW were subsequent community SSE clusters that had a strong putative environmental etiology (viz., sewage pipes, building design, and poor environmental hygiene) in addition to human-to-human transmission [Hong Kong Special Administrative Region (HKSAR) 2003; Wong and Hui 2004]. As would be expected because of a large patient catchment area, the PWH cluster was more geographically widespread (as supported by the SD ellipses in Figure 6D) compared with the AMOY cluster (Figure 6B), the sample size of which was one-third larger. The SD ellipses of the PWH cluster (Figure 6D) reveal a northwest–southeast directional trend of disease spread that extends over most of Hong Kong. The AMOY cluster was comparatively more localized, and the map had to be be enlarged to show the standard ellipses that exhibit an almost east–west directional trend of disease transmission (Figure 6B). The NTKLOW cluster (Figure 6F) was the least geographically widespread of the three SSEs, where the very compact spatial distribution must be magnified to visualize details of the SD ellipses.

**Figure 7:**
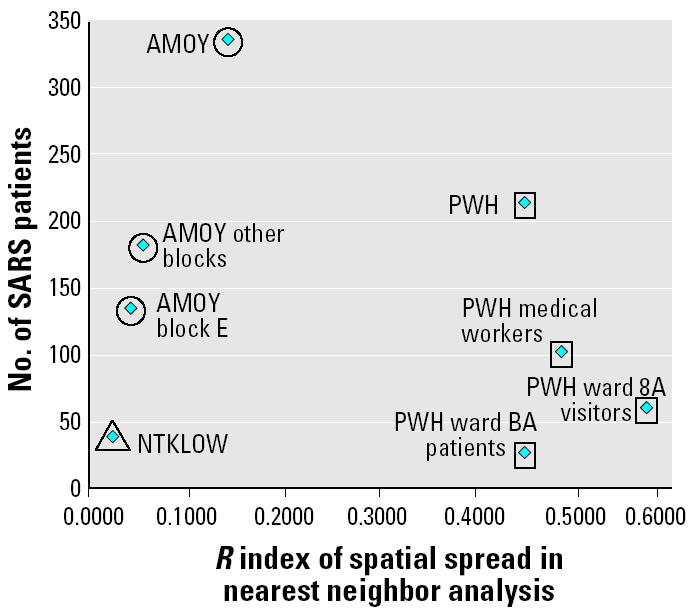
Spatial clusters of SARS patients (February–June 2003) by nearest neighbor analysis.

Figure 7 and Table 4 show low *R* scores (a measure to inform the extent of disease spread) indicating a high degree of clustering for all three SSEs. The *R* values were significant at the 0.001 level, confirming that the point patterns exhibited a tendency toward clustering. Figure 7 also shows that block E of AMOY (the epicenter of the AMOY SSE), with a lower *R* score, exhibited a more compact geospatial arrangement in SARS infection than did other apartment blocks within AMOY. Visitors of ward 8A (the epicenter of the PWH SSE where the index patient of the cluster stayed) of the PWH were found to spread the disease farthest from its source of all the three clusters examined here, as would be expected for such a nosocomial outbreak at a tertiary referral hospital where SARS patients were densely aggregated on the ward but visiting relatives and friends returned home situated in different parts of Hong Kong (and not necessarily from the immediate surrounding neighborhood, given that the hospital is one of only two tertiary referral centers in the territory with a very wide catchment area). The NTKLOW cluster recorded the lowest *R* score, substantiating earlier observations from Figure 6.

## Discussion

Our findings show that GIS methods can be usefully employed during an acute infectious disease outbreak to reveal new geospatial information in addition to standard field epidemiologic analyses. This mapping and cartographic technique can provide visual display of information in both space and time simultaneously. When applied in real time during the onset and evolution of an epidemic, it can monitor and enhance understanding of the transmission dynamics of an infectious agent, thereby facilitating the design, implementation, and evaluation of potential intervention strategies. GIS can offer quantitative and statistical measures along with visualization tools to examine patterns of disease spread with respect to disease clusters. Disease mapping is a first step toward understanding spatial aspects of health-related problems, as particular kinds of information are highlighted in maps. Various cartographic symbolizations (as points, lines, or areal patterns) can show the distribution of diseases. Disease clusters and other associations can then be deduced statistically and visually after examining the disease maps. In Chomsky’s (1965) terms, analyses at the first two levels concern the surface structure of an event, whereas the third level seeks to extract deep structure information. Surface structure information is simple and immediately perceptible to a user, whereas deep structure information is content-specific knowledge needed for problem solving (Nyerges 1991).

In the case of SARS in Hong Kong, our study, first and foremost, demonstrates exceptional spatial clustering of the cases. The kernel method adjusted for population density provided a means of highlighting population at risk, whereas the use of *R* values and Moran’s coefficients in conjunction with map displays enhanced the analytical context of the point pattern distributions. In fact, such geospatial intelligence gathered from examining statistical surfaces and disease clusters provided the basis for the formulation of our transmission dynamics model (Riley et al. 2003). More specifically, choice of a suitable framework was not straightforward in constructing the transmission dynamics model where a variety of approaches were possible, ranging from a simple deterministic compartmental approach to a spatially explicit, individual-based simulation. Given the data available for Hong Kong, we based our analyses on a stochastic metapopulation compartmental model. A metapopulation approach was appropriate because the incidence of SARS varied substantially by geographical district, as the GIS analyses have shown.

Second, the simultaneous geospatial–temporal approach to modeling the SARS outbreak revealed complementary additional information that would otherwise not be available from the traditional epidemic curve method (a standard public health outbreak investigative approach) in identifying the mode of spread. The daily animated series of kernel maps clearly shows that SARS was a highly localized disease; thus, its route of transmission was unlikely to be through casual contact, as it is for influenza and measles, but more compatible with close contact via heavy respiratory droplets and fomites. This confirms that SARS is only a moderately transmissible condition with a basic reproduction number of about 3 (Riley et al. 2003), in contrast to measles and influenza, which have basic reproduction numbers of about 13 and 5, respectively (Anderson and May 1991; Ferguson et al. 2003). An alternative interpretation of the observed high degree of geospatial clustering would be that SARS was due to an environmental point source outbreak. Indeed, faulty sewage systems and the “chimney effect” is the leading hypothesis explaining the AMOY SSE (especially block E), although some have suggested roof rats as a vector (Ng 2004). Although it is difficult to gauge retrospectively, had the GIS system we implemented in this report been available for near real-time analysis, it would likely have detected the highly unusual clustering of cases in SSEs such as the PWH and AMOY outbreaks much sooner, as they evolved. This in turn could have resulted in more rapid contact tracing and public health intervention, thus perhaps mitigating the extent of spread substantially in the case of person-to-person transmission events and preventing further large-scale environmental point source outbreaks in residential apartment blocks (although it would not have made a difference to AMOY itself given the temporally abrupt and short-lived environmental release of viral particles).

Third, contextual analysis of mean and SD values of different density classes, particularly after logarithmic transformation to accentuate near zero values on a graph, provided a geographic approach to estimating the beginning and subsidence of a large degree of spread of SARS in the community. This is a useful adjunct to the usual biomathematical modeling approach using reproductive numbers at different points in time, representing the average number of infections, excluding SSEs, caused by infected individuals in successive generations at time *t* throughout the SARS epidemic (Riley et al. 2003).

Fourth, the SD ellipses from the OD analysis, coupled with complementary results from *R* and Moran’s *I* values, yielded information on the direction of spread in a disease cluster that can be used to inform contact tracing and the design of quarantine measures. In the case of SARS in China, where entire residential districts were cordoned off for weeks at the height of the outbreak, the selection of such districts for quarantine could have been better informed by these ellipses indicating directional bias and associated physical distance in disease transmission.

There are, however, limitations and caveats to the GIS technique in infectious disease epidemiology and outbreak investigation. Howe (1963) argued that mapping of diseases tended to expose the “where” but not “why there” of the outbreak. Nevertheless, elementary descriptive analysis as an output of disease mapping can be a source of new leads for further exploratory analyses. Map patterns can provide stimuli for generating hypotheses of disease causation (Lloyd and Yu 1994; McKee et al. 2000). Moreover, newer developments that complement traditional mapping functions such as cluster and contextual analyses can be very useful adjunct investigative tools in outbreak control, as our example on SARS in Hong Kong has highlighted.

The completeness and availability of necessary data are another area of potential concern where conventional field epidemiologic data collection forms rarely contain the full range of variables that are required in a GIS analysis. Data consistency and, in particular, the nonstandardization of patient address formats is one such example. Field epidemiologists often relegate certain personal particulars such as residential and work addresses to a lower priority in their data collection procedures, or at least enter the information in a haphazard fashion, rendering GIS analysis very difficult by diminishing the proportion of usable cases for analyses. Similar generic problems that plague the establishment of all information systems must be resolved to enable real-time disease monitoring and surveillance. They include lack of standardization for data capture documents, procedures and protocols for information management, delays in transferring and updating information, and a lack of rapid analysis and audit of databases. The SARS epidemic is a clear signal that Hong Kong needs much greater and sustained investment in health informatics, that is, public health information systems, the skills to use them, and networks to share them.

In summary, integration of GIS technology into routine field epidemiologic surveillance can offer a scientifically rigorous and quantitative method for identification of unusual disease patterns in real time, as our example of SARS has shown. Its potential can be synergistically maximized when linked with clinical databases collecting data at the point of care across the whole population as well as environmental data sources (e.g., meteorologic, transportation, topographical information) to rapidly recognize, locate, and monitor disease outbreaks.

## Figures and Tables

**Table 1:** Urban area and population data of Hong Kong by districts.

18 Districts plus marine	Total population	Total working population*^a^*	Percent urban land*^b^*	Percent urban allocation*^c^*
Central and western	261,884	144,824	29	99.9
Eastern	616,199	314,674	27	99.7
Islands	86,667	43,201	1	97.8
Kowloon City	381,352	185,553	82	99.9
Kwai Tsing	477,092	218,291	44	99.9
Kwun Tong	562,427	226,062	76	99.9
North	298,657	133,767	12	99.1
Sai Kung	327,689	165,219	3	99.8
Shatin	628,634	265,473	17	99.9
Sham Shui Po	353,550	159,861	53	99.9
Southern	290,240	145,086	9	98.9
Tai Po	310,879	145,520	6	99.6
Tsuen Wan	275,527	140,011	8	99.9
Tuen Mun	488,831	210,115	17	99.6
Wan Chai	167,146	93,365	33	99.9
Wong Tai Sin	444,630	200,265	47	99.9
Yau Tsim Mong	282,020	137,765	64	99.9
Yuen Long	449,070	180,198	21	99.1
Marine*^d^*	5,895	4,629	0	20.7
Total	6,708,389	3,113,879		

Data from Hong Kong Census and Statistics Department (2002).

a Sum of employed labor force.

b Total urban areas within each district divided by district area.

c Computed from urban-related occupation in employed labor force, defined as follows: rural-related occupation (includes agriculture and fishing); mining and quarrying; urban-related occupation (includes community, social, and personal services); construction; electricity, gas, and water; financing; insurance, real estates and business services; manufacturing; transport, storage, and communications; wholesale, retail, and import/export trades; restaurants and hotels; unclassified.

d Marine data were not land based and thus were excluded from the study.

**Table 2:** A frequency breakdown of SARS-infected buildings (February–June 2003).

No. of SARS cases in a building	No. of buildings	Total no. of SARS cases
136	1	136
47	1	47
46	1	46
43	1	43
20	1	20
18	1	18
11	1	11
10	3	30
9	1	9
8	3	24
7	2	14
6	6	36
5	3	15
4	12	48
3	47	141
2	156	312
1	759	759
		Total 1,709

**Table 3:** Mean and SD of infection rates of 12 prototypical days.

	Day 1 (18 Feb)	Day 16 (6 Mar)	Day 20 (10 Mar)	Day 22 (12 Mar)	Day 28 (18 Mar)	Day 38 (28 Mar)	Day 40 (30 Mar)	Day 48 (7 Apr)	Day 56 (15 Apr)	Day 67 (26 Apr)	Day 79 (8 May)	Day 106 (4 Jun)
No. of patients	1	15	107	120	126	421	276	187	129	67	29	2
Mean	0.020	0.023	0.041	0.046	0.047	0.151	0.108	0.066	0.046	0.031	0.026	0.020
*z*0.01 = 2.33	0.00000	0.43580	0.75503	0.73118	0.74243	0.22811	0.24205	0.64763	0.70296	0.63733	0.52928	0.09170
SD	0.009	0.023	0.106	0.138	0.137	1.852	1.202	0.279	0.129	0.057	0.047	0.003
*F*0.01(14,14) = 3.6	1.00	20.00*	503.86*	703.10*	759.91*	102817.04*	38521.60*	2277.52*	873.57*	250.41*	58.85*	2.05

*p < 0.001 indicates that the null hypothesis is rejected and that the SD is significantly different from or greater than that of day 1. *z* = 2.33 and *F*(14,14) = 3.6 at the 0.01 level of significance for one-tailed tests.

**Table 4:** Index of spatial spread by nearest neighbor analysis.

Description	*R*	*n*
AMOY cluster	0.15*	335
AMOY block E residents	0.05*	132
AMOY block E visitors		3
Other block residents	0.06*	181
Other block visitors		5
Visited AMOY shopping mall		14
PWH cluster	0.45*	212
PWH		18
Ward 8A visitors	0.58*	58
Ward 8A patients	0.45*	25
PWH medical workers	0.49*	99
PWH other		12
NTKLOW cluster	0.02*	38

*n*, number of SARS patients.

*p < 0.001 indicates that the null hypothesis is rejected; a tendency towards clustering exists.

## References

[b1-ehp0112-001550] Affonso DD, Andrews GJ, Jeffs L (2004). The urban geography of SARS: paradoxes and dilemmas in Toronto’s health care. J Adv Nurs.

[b2-ehp0112-001550] Anderson RM May RM 1991. Infectious Diseases of Humans: Dynamics and Control. Oxford:Oxford University Press.

[b3-ehp0112-001550] Bailey TC Gatrell AC 1995. Interactive Spatial Data Analysis. Essex, UK:Longman Group. 10.1016/0277-9536(95)00183-2 8778997

[b4-ehp0112-001550] Batten DF Boyce DE 1986. Spatial interaction, transportation, and interregional commodity flow models. In: Handbook of Regional and Urban Economics, Vol 1 (Nijkamp P, ed). Amsterdam:North Holland, 357–406.

[b5-ehp0112-001550] Chomsky N 1965. Aspects of the Theory of Syntax. Cambridge, MA:MIT Press.

[b6-ehp0112-001550] Cliff AD Haggett P 1988. Atlas of Disease Distributions—Analytic Approaches to Epidemiological Data. Oxford, UK:Basil Blackwell.

[b7-ehp0112-001550] Cui Y Zhang ZF Froines J Zhao J Wang H Yu SZ 2003. Air pollution and case fatality of SARS in the People’s Republic of China: an ecologic study. Environ Health 2(1):15. Available: http://www.ehjournal.net/content/2/1/15 [accessed 10 June 2004]. 10.1186/1476-069X-2-15 PMC293432 14629774

[b8-ehp0112-001550] Donnelly CA Ghani AC Leung GM Hedley AJ Fraser C Riley S 2003. Epidemiological determinant of spread of causal agent of severe acute respiratory syndrome in Hong Kong. Lancet 361:1761–1766. Available: http://image.thelancet.com/extras/03art4453web.pdf [accessed 15 May 2004]. 10.1016/S0140-6736(03)13410-1 PMC7112380 12781533

[b9-ehp0112-001550] Eck JE Weisburd D eds. 1995. Crime and Place, Crime Prevention Studies, Vol 4. Monsey, NY:Criminal Justice Press.

[b10-ehp0112-001550] Ferguson NM, Galvani AP, Bush RM (2003). Ecological and immunological determinants of influenza evolution. Nature.

[b11-ehp0112-001550] Getis A, Ord JK (1992). The analysis of spatial association by use of distance statistics. Geogr Anal.

[b12-ehp0112-001550] Haggett P (1994). Geographical aspects of the emergence of infectious diseases. Geografiska Annaler. Hum Geogr Chang Geogr Dis Distr.

[b13-ehp0112-001550] Higgs G, Gould M (2001). Is there a role for GIS in the “new NHS”?. Health Place.

[b14-ehp0112-001550] HKSAR 2003. SARS in Hong Kong: From Experience to Action. Report of SARS Expert Committee. Hong Kong:Hong Kong Special Administrative Region. Available: http://www.sars-expertcom.gov.hk/english/reports/reports/reports_fullrpt.html [accessed 14 September 2004].

[b15-ehp0112-001550] Hong Kong Census and Statistics Department 2002. Hong Kong 2001 Population Census—TAB on CD-ROM and MAP on CD-ROM. Hong Kong:Census and Statistics Department.

[b16-ehp0112-001550] Howe GM 1963. National Atlas of Disease Mortality in the United Kingdom. London:T. Nelson.

[b17-ehp0112-001550] Kafadar K (1996). Smoothing geographical data, particularly rates of disease. Stat Med.

[b18-ehp0112-001550] Krebs CJ 1989. Ecological Methodology. New York:Harper and Row.

[b19-ehp0112-001550] Lai PC Chan K 2004. Kernel Density Estimation of Temporal Changes of SARS Cases in Hong Kong (with 5-Day Incubation). Hong Kong:Department of Geography, University of Hong Kong. Available: http://geog.hku.hk/pclai/kernel/ (username: kernel; password: flash) [accessed 15 May 2004].

[b20-ehp0112-001550] Lau JT, Fung KS, Wong TW, Kim JH, Wong E, Chung S (2004). SARS transmission among hospital workers in Hong Kong. Emerg Infect Dis.

[b21-ehp0112-001550] Leung GM Hedley AJ Ho LM Chau P Wong IOL Thach TQ In press. The epidemiology of severe acute respiratory syndrome (SARS) in the 2003 Hong Kong epidemic: analysis of all 1,755 patients. Ann Intern Med. 10.7326/0003-4819-141-9-200411020-00006 15520422

[b22-ehp0112-001550] Lloyd OL, Yu TS (1994). Disease mapping: a valuable technique for environmental medicine. J Hong Kong Med Assoc.

[b23-ehp0112-001550] McKee KT, Shields TM, Jenkins PR, Zenilman JM, Glass GE (2000). Application of a geographic information system to the tracking and control of an outbreak of Shigellosis. Clin Infect Dis.

[b24-ehp0112-001550] Meade MS Earickson RJ 2000. Medical Geography. London:Guilford Press.

[b25-ehp0112-001550] Ng S 2004. The mystery of Amoy Gardens. In: At the Epicentre: Hong Kong and the SARS Outbreak (Loh C, ed). Hong Kong:University of Hong Kong Press, 95–116.

[b26-ehp0112-001550] Nyerges TL (1991). Analytical map use. Cartogr Geogr Inf Syst.

[b27-ehp0112-001550] Olson J (1976). A coordinate approach to map communication improvement. Am Cartogr.

[b28-ehp0112-001550] Riley S, Fraser C, Donnelly CA, Ghani AC, Abu-Raddad LJ, Hedley AJ (2003). Transmission dynamics of the etiological agent of severe acute respiratory syndrome (SARS) in Hong Kong: the impact of public health interventions. Science.

[b29-ehp0112-001550] Sawada M 2001. Global Spatial Autocorrelation Indices—Moran’s *I*, Geary’s *C* and the General Cross-Product Statistic. Ottawa, Ontario, Canada:Department of Geography, University of Ottawa. Available: http://www.uottawa.ca/academic/arts/geographie/lpcweb/newlook/publs_and_posters/reports/moransi/moran.htm#top [accessed 10 June 2004].

[b30-ehp0112-001550] So FM 2002. An Application of Geographic Information Systems in the Study of Spatial Epidemiology of Respiratory Diseases in Hong Kong, 1996–2000 [MPhil thesis]. Hong Kong:University of Hong Kong.

[b31-ehp0112-001550] Song C Kulldorff M 2003. Power evaluation of disease clustering tests. Int J Health Geogr 2(9). Available: http://www.ij-healthgeographics.com/content/pdf/1476-072X-2-9.pdf [accessed 10 June 2004]. 10.1186/1476-072X-2-9 PMC333429 14687424

[b32-ehp0112-001550] Taylor PJ 1977. Quantitative Methods in Geography—An Introduction to Spatial Analysis. Boston:Houghton Mifflin.

[b33-ehp0112-001550] WHO 2003. Consensus Document on the Epidemiology of Severe Acute Respiratory Syndrome (SARS). WHO/CDS/CSR/GAR/2003.11. Geneva:World Health Organization. Available: http://www.who.int/csr/sars/en/WHOconsensus.pdf [accessed 15 May 2004].

[b34-ehp0112-001550] Wong RS, Hui DS (2004). Index patient and SARS outbreak in Hong Kong. Emerg Infect Dis.

